# Hidden bias in the DUD-E dataset leads to misleading performance of deep learning in structure-based virtual screening

**DOI:** 10.1371/journal.pone.0220113

**Published:** 2019-08-20

**Authors:** Lieyang Chen, Anthony Cruz, Steven Ramsey, Callum J. Dickson, Jose S. Duca, Viktor Hornak, David R. Koes, Tom Kurtzman

**Affiliations:** 1 Department of Chemistry, Lehman College, Bronx, New York, United States of America; 2 Ph.D. program in Biochemistry, The Graduate Center of the City University of New York, New York, United States of America; 3 Ph.D. program in Chemistry, The Graduate Center of the City University of New York, New York, United States of America; 4 Computer-Aided Drug Discovery, Global Discovery Chemistry, Novartis Institutes for Biomedical Research, Cambridge, Massachusetts, United States of America; 5 Department of Computational and System Biology, University of Pittsburgh, Pittsburgh, Pennsylvania, United States of America; University of Michigan, UNITED STATES

## Abstract

Recently much effort has been invested in using convolutional neural network (CNN) models trained on 3D structural images of protein-ligand complexes to distinguish binding from non-binding ligands for virtual screening. However, the dearth of reliable protein-ligand x-ray structures and binding affinity data has required the use of constructed datasets for the training and evaluation of CNN molecular recognition models. Here, we outline various sources of bias in one such widely-used dataset, the Directory of Useful Decoys: Enhanced (DUD-E). We have constructed and performed tests to investigate whether CNN models developed using DUD-E are properly learning the underlying physics of molecular recognition, as intended, or are instead learning biases inherent in the dataset itself. We find that superior enrichment efficiency in CNN models can be attributed to the analogue and decoy bias hidden in the DUD-E dataset rather than successful generalization of the pattern of protein-ligand interactions. Comparing additional deep learning models trained on PDBbind datasets, we found that their enrichment performances using DUD-E are not superior to the performance of the docking program AutoDock Vina. Together, these results suggest that biases that could be present in constructed datasets should be thoroughly evaluated before applying them to machine learning based methodology development.

## Introduction

Virtual screening plays an essential role in lead identification in the early stages of drug discovery [1,2]. Accurate lead identification can dramatically reduce the time and costs associated with experimental assays. Therefore, developing computational tools that can identify lead compounds with pharmacological activity against a selected protein target has been a long-standing goal for computational chemists. A number of structure-based docking tools that aim to predict ligand binding poses and binding affinities have been developed and have enjoyed moderate success over the last three decades [3–12].

Inspired by the success that deep learning has achieved in speech and image recognition [13–18], many groups have sought to apply deep learning methodology to protein-ligand binding prediction [19–27]. Of these, the grid-based CNN approach has been reported to have promising performance [21,25–27]. The approach constructs a 3D grid of atom type densities from the protein-ligand structure in the binding site. When training a virtual screening model, these grids are fed into the model, which automatically optimizes its parameters to minimize a loss function whose value reflects the model’s ability to distinguish between binding and non-binding compounds in the training set.

While CNN algorithms have existed for some time [28,29], the recent resurgence and success of CNN-based methods has widely been attributed to increased computational power and the development of large, highly-curated datasets [18]. It is generally believed that in order to implement CNN-based models in virtual screening, large and diverse training sets and independent test sets are required to effectively train and objectively evaluate the models [30].

The Database of Useful Decoys-Enhanced (DUD-E) contains a large number of experimentally verified actives and property-matched decoys and has been widely utilized to train and test machine learning models and compare their performance with that of simple docking tools [23–25,31–37]. In many CNN-based virtual screening studies, it is typical to see models achieve an area under the receiver operating characteristic (ROC) curve (AUC) greater than 0.9 for many targets from DUD-E [22,25,27]. Although some studies have indicated that DUD-E may have limited chemical space and issues with analogue bias and bias resulting from the decoy compound selection criteria [38,39], it has not been clearly elucidated how these potential biases affect CNN model development and performance.

A perceived advantage of CNN-based virtual screening approaches over more traditional approaches such as physics-based empirical scoring is that, rather than requiring manual tuning of weights and terms of a scoring function, CNN models can automatically learn the features that determine binding affinity between a ligand and its protein target. However, the main disadvantage of complex machine learning models such as CNN is that it is unclear what features of a dataset the model is prioritizing in making its binding assessments. In a traditional parameterized scoring function, each term has a physically-meaningful interpretation (H-bond and hydrophobic contacts, ligand desolvation, etc.) and the importance of each term can be assessed by their relative weights. In machine learning approaches, there are no such easily-interpretable terms, and it is difficult to assess what the models are actually learning.

To investigate the causes that lead to the high performance of CNN-based virtual screening, we define three sources of information that the models can learn from. **1) Protein-ligand interactions**: It is widely believed that the physics that govern molecular recognition will apply to novel targets and drug candidates. A hope for the machine learning-based approach is that models will learn the essential physics of molecular interactions and therefore be applicable to new targets and the exploration of a novel ligand chemical space. **2) Analogue bias**: Binders of the same target, homologous targets, or targets with similar functionality are thought to be correlated in chemical space. Models that learn these correlations could be applied to find additional compounds that are similar to existing known binders of such targets. **3) Decoy bias**: For each target in DUD-E, decoys were selected by the authors with the criteria that the decoy ligands have similar physical properties to the actives but differ topologically. However, this might lead to the decoys being distinguishable from the actives by patterns resulting from the selection criteria. A model that learns such patterns can distinguish decoys from actives only when the decoys fit the biased feature pattern and would likely not be applicable to the prospective identification of novel compounds.

In the following work, we carefully construct training and test set combinations that are designed to isolate or minimize the contributions of each of these biases. We find that the high performance of CNN models trained on DUD-E is not attributable to having learned the features of protein-ligand interactions but rather to analogue and decoy bias inherent in the DUD-E dataset. We show that it is incorrect to infer that a model has successfully learned protein-ligand interactions solely on the basis of its high performance on a test set. Due to the hidden biases in the DUD-E dataset that we describe in this work, one should be very cautious when using DUD-E for machine learning based methodology development.

## Methods

### Preparation of model input data

The CNN model requires as input ligands posed in a protein-binding pocket with each ligand marked as active or inactive. In this work, we used the complete set of proteins from the DUD-E dataset, which is one of the most widely-used datasets used to develop and validate virtual screening approaches. The dataset consists of 102 targets, each of which has a group of experimentally-tested active molecules and property-matched decoys. In total, it contains 22,886 actives and over a million decoys [37].

Most of the actives in DUD-E do not have crystal binding poses. We generated poses for all the actives and decoys in the training and test sets using the smina implementation of AutoDock Vina [10,40]. All compounds are docked against the reference receptor within an 8 Å cubic box centered around a reference ligand. The docked data can be found at http://bits.csb.pitt.edu/files/docked_dude.tar. In this study, only the top-ranking pose as scored by Vina for each active and decoy was used as input for the CNN model.

### Training and test set preparation

Training and test subsets of the DUD-E dataset were constructed in several different ways.

#### Single target CNN model

**Fig 1:**
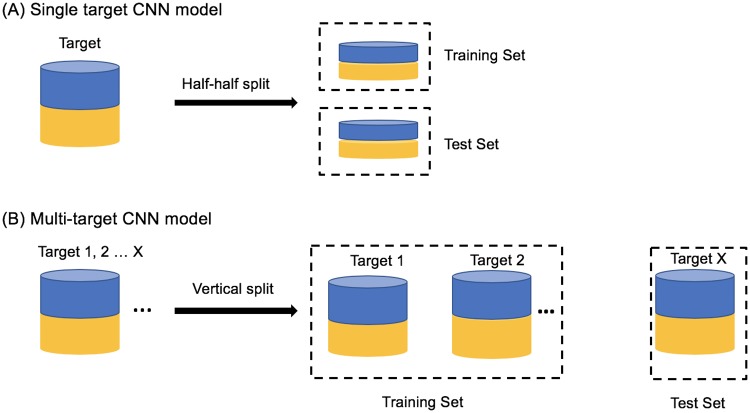
Data preparation for model training and testing. The training set and test set for (A) the singe target CNN model and (B) multi-target CNN model. Blue denotes actives and yellow denotes decoys.

To build the single target CNN model, for each target, we randomly selected half of the actives for training and used the remaining half for model evaluation. To reduce the training time and partially compensate for the imbalance in the number of actives and decoys, for each target, we randomly selected 1000 decoys and used 500 for training and 500 for testing. See Fig 1A.

#### Multi-target CNN model

To build the multi-target CNN model, we trained on a subset of protein targets and tested on the remaining protein targets. The models were trained on half of the actives and 500 randomly-selected decoys for each target in the training subset. See Fig 1B.

#### Actives as decoys dataset

The Actives as Decoys (AD) dataset was designed to minimize the decoy bias introduced by the selection criteria in the construction of the DUD-E dataset. Instead of using the DUD-E decoys, the AD (actives as decoys) dataset uses the active compounds of other proteins as the decoys for each target. The DUD-E dataset is composed of 102 targets each of which has a set of active and inactive compounds. For each protein target, we docked (using Vina) all of the active compounds from the other 101 DUD-E proteins to that target. For example, for target AA2AR we took the actives of each of the other 101 proteins from the DUD-E dataset (ABL1, ACE, ACES, …) and docked them to AA2AR. For each of the 101 proteins, we rank-ordered their respective actives based on their predicted binding affinity (returned by Vina) to the target (AA2AR in this case) and chose the top 50 compounds. We compiled these compounds to create a decoy dataset for the target (AA2AR in this case). If the number of actives for a protein was less than 50, then all compounds were used. The AD dataset for all 102 targets can be downloaded here (www.lehman.edu/faculty/tkurtzman/files/102_targets_AD_dataset.tar).

### Model training

#### CNN model

**Fig 2:**
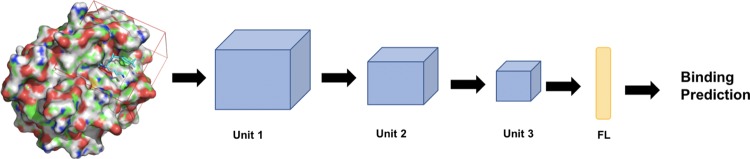
The architecture of the CNN model. Each unit consists of three layers, Pooling, Convolutional and ReLU. The yellow bar labeled FL is the fully connected layer. Further details about the CNN model hyperparameters can be found in reference [41].

Our CNN models were defined and trained by the Caffe deep learning framework; the model architecture is as previously described [41]. The source code can be found at https://github.com/gnina/gnina. Briefly, the binding complex is transformed into a grid of atomic densities. The grid is 24 Å per side and composed of 48 * 48 * 48 voxels in 0.5 Å resolution centered on the ligand binding site. Each voxel has 39 channels in total: 35 channels of atom density information corresponding to 16 protein atom types, 19 ligand atom types (S1 Table), and, optionally, 4 channels for water thermodynamic information computed by GIST [42]. Water thermodynamic information was not part of the originally published CNN model. It was added here to explore whether adding solvation effects to the protein-ligand system improves the performance of the CNN model. We built three kinds of CNN models: 1) receptor-ligand-water model, 2) receptor-ligand model and 3) ligand-only model, distinguished by the binding information used for model training. The receptor-ligand-water model uses all 39 channels of information, and the receptor-ligand model uses just the information from the 35 atomic densities. In the ligand-only model, the original receptor is replaced by a single dummy atom; therefore, the atomic density values from the 16 receptor channels all equal zero, and only the 19 channels from the ligand are used. As illustrated in Fig 2, the input tensor that consists of a specific number of channels plus a label of 1 denoting an active compound or 0 for an inactive compound is then fed to the model, which consists of three units of Pooling (2*2*2 filter)- Convolutional (3*3*3 filter)-ReLU layers and a single fully-connected layer that outputs the binding prediction. During training, we used a learning rate of 0.01, a momentum of 0.9, an inverse learning rate decay with power = 1 and gamma = 0.001, and a weight decay of 0.001. In each training iteration, we used balanced actives and decoys with a batch size of 10 for 2000 iterations. We manually checked that all models qualitatively converged at the end of the training. The protocol for training the CNN model can be found here (https://www.protocols.io/view/train-cnn-model-using-gnina-3rngm5e).

#### K-nearest neighbors (KNN) model

KNN classification predicts a ligand’s label (binder or nonbinder) based on the majority vote of its K nearest neighbors in a defined feature space. Here, the input for the KNN model were “RDKit” fingerprints (2D) generated by the RDKit python package (http://www.rdkit.org, version 2018.09.1). The bit size for the “RDKit” fingerprints is 2048, and the script that was used to output the fingerprints can be found at https://github.com/dkoes/qsar-tools/blob/master/outputfingerprints.py. To compare the KNN model’s performance with that of the ligand-only CNN model, the same training and test sets were used for each target. The python scripts we used for training KNN models can be found here: https://github.com/dkoes/qsar-tools.

#### Grid inhomogeneous solvation theory (GIST)-based water analysis

**Fig 3:**
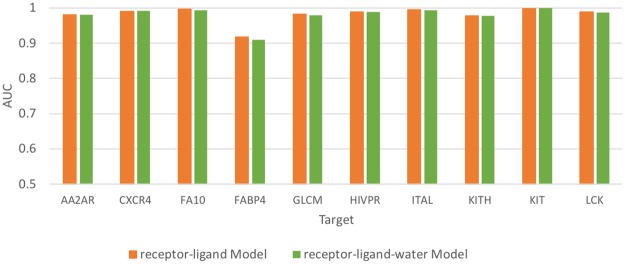
The performance of receptor-ligand and receptor-ligand-water CNN models in 10 DUD-E targets.

To investigate whether adding water information to the protein-ligand binding complex could improve the accuracy of binding prediction, we applied GIST from AmberTools to map out the water properties by analyzing the water trajectory produced by molecular dynamic (MD) simulation [42,43]. Protein structures were downloaded from the Protein Databank [44] and proteins were prepared using the default parameters in the Maestro Protein Preparation Wizard (Schrödinger) [45]. As we were interested in the solvation of the binding sites, membranes were not modeled for trans-membrane proteins as they were distal to the active site. The MD simulations were conducted with Amber16 using the ff14SB forcefield [46–48]. A subset of prepared apo-proteins, listed in Fig 3, were placed in a box of OPC water such that all protein atoms were at least 10 Å from the periodic boundary of the box. The equilibration run consisted of two minimizations of up to 20,000 cycles followed by a 240 ps run at constant volume where the temperature of the simulations was raised from 0 to 300 K and protein heavy atoms were harmonically restrained with a force constant of 100 kcal/mol•Å^2^. Next, we performed an additional equilibration MD run of 20 ns under NPT conditions with the 100 kcal/mol•Å^2^ gradually reduced to 2.5 kcal/mol•Å^2^ in the first 10 ns and held constant for the last 10 ns. Production simulations were then performed for 100 ns in NVT conditions at 300 K, with heavy atom restraints of 2.5 kcal/mol•Å^2^. The 100 ns trajectories were then processed by AmberTools cpptraj-GIST with a grid spacing of 0.5 Å^3^, centered on the ligand binding sites to produce solvation thermodynamic maps. The resulting GIST maps of the solute-water enthalpy (E_sw_), water-water enthalpy (E_ww_), translational entropy (TS_trans_), and orientational entropy (TS_orient_) were added as the 4 additional channels to the original 35 protein-ligand channels to train the protein-ligand-water models.

## Results

### Adding water information does not improve the performance of the protein-ligand CNN model

Using the single target CNN model approach, we independently trained the protein-ligand and protein-ligand-water CNN models on 10 targets from the DUD-E dataset. Originally, we hypothesized that adding water information channels could improve virtual screening performance as shown in previous work by Balius *et al*., in which adding water energy terms to scoring functions improved the virtual screening performance of DOCK3.7 [5]. As shown in Fig 3, the receptor-ligand CNN model achieved high enrichment efficiency (0.98 ± 0.02), which is consistent with the results from other studies using the CNN approach [25,27]. Given that the AUC in the protein-ligand models was already high, adding the water channels resulted in no detectable increase in the test set AUC.

### Performances of the receptor-ligand and ligand-only CNN models are equivalent

**Fig 4:**
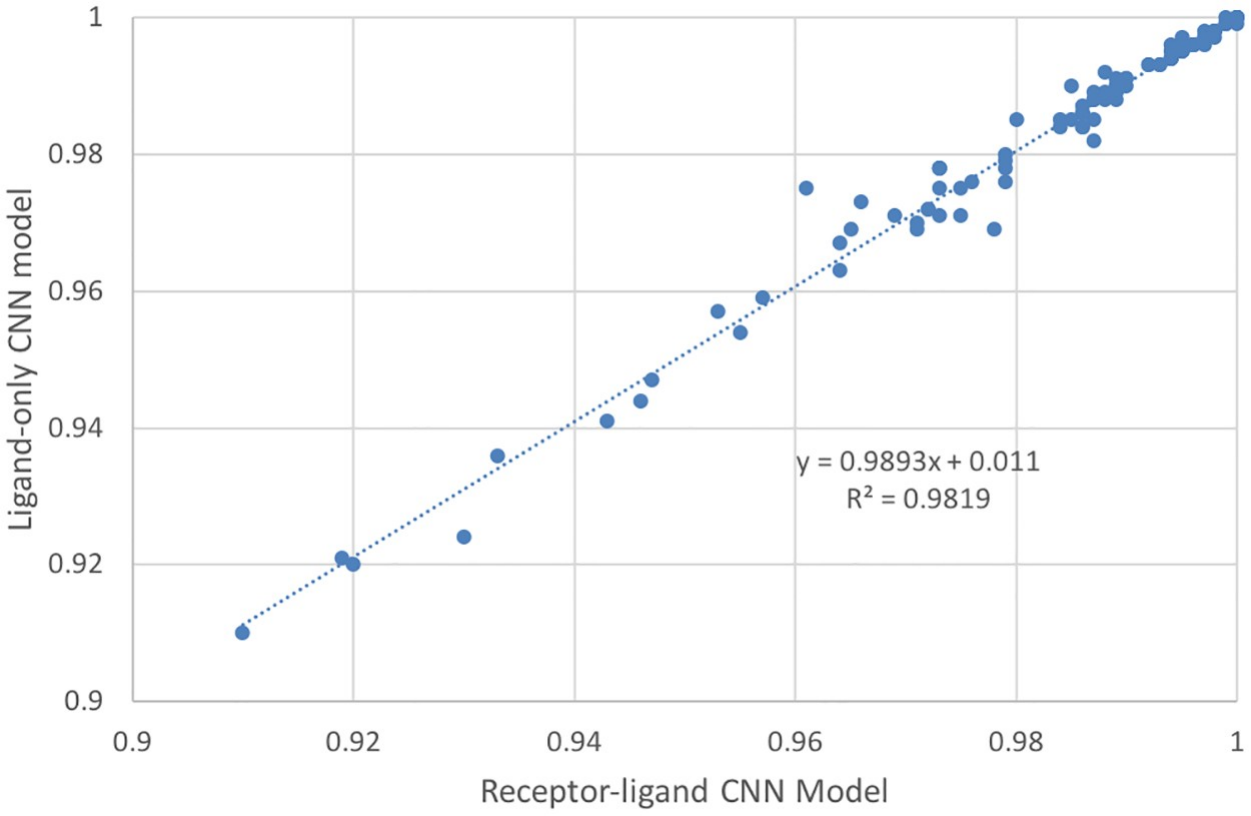
Correlation between the performance of the receptor-ligand CNN model and ligand-only CNN model. The receptor-ligand CNN model was trained on receptor-ligand 3D binding poses, and the ligand-only CNN model was trained on ligand binding poses alone. Each blue dot is a target from DUD-E; there are 102 targets in total.

Given the high AUC achieved by receptor-ligand models, we were interested in whether these models have successfully learned from the protein-ligand molecular interactions or were instead learning from ligand bias. To test this, we built two single-target CNN models for each DUD-E target: the receptor-ligand model and the ligand-only model. The receptor-ligand model was trained on the receptor-ligand 3D binding pose, while in the ligand-only model, each receptor structure was replaced by a single identical dummy atom. The model was therefore trained by the ligand binding pose alone without any meaningful receptor information. Strikingly, as shown in Fig 4, the AUC values achieved by the receptor-ligand model and ligand-only model were highly correlated (R^2^ = 0.98, slope K = 0.99). Both the receptor-ligand model and ligand-only model achieved an average AUC of 0.98, with AUC greater than 0.9 for all 102 DUD-E targets. The average absolute difference in the AUC values of the two types of models for the 102 targets was 0.001. This suggests that the CNN algorithm can determine a set of parameters to accurately distinguish the actives from the decoys for a specific target regardless of whether the receptor structure is provided or not.

### The receptor-ligand model does not learn from protein structure information

**Fig 5:**
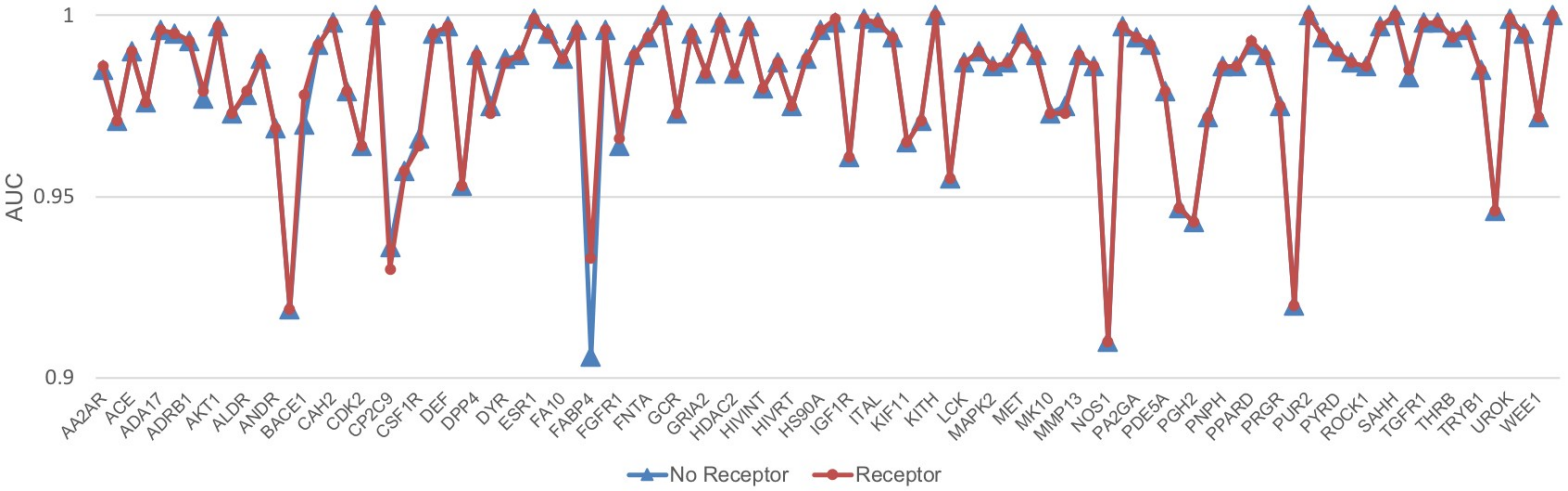
Performance of the receptor-ligand model for the same ligand test sets with and without receptor information. For each target, red dots indicate performance when the receptor structure was provided in the test set, while blue triangles indicate performance when the receptor structure was replaced by a single dummy atom. The x-axis displays each DUD-E target in the same order as they appear in the DUD-E database (http://dude.docking.org/targets). The targets with even indices are not labeled on the x-axis due to space limitations.

Given the ligand model’s high performance, we were interested in determining how much the receptor structure contributed to the receptor-ligand model’s performance. To test this, we used the same receptor-ligand model trained as above on the receptor and ligand information and then tested it on two datasets. The first dataset input all the appropriate structural information into the channels for both the receptor and ligand. The second testing dataset used all the ligand structure information but replaced the receptor structure information with information for a single dummy atom, thereby providing no protein structure information. The results of these tests are shown in Fig 5. Surprisingly, the receptor-ligand models performed almost exactly the same regardless of whether information on the receptor was provided in the test set. The average AUC for both datasets is 0.98, and the average absolute AUC difference between the two testing sets is 0.0006, with the largest difference (0.027) for FABP4. This strongly suggests that the receptor-ligand model is learning almost entirely from the ligand information and not from receptor-ligand binding patterns. It is generally thought that CNN algorithms will use all the information from the input to optimize the model parameters. Strikingly, here, we show that for almost all targets, only the ligand information was necessary for the receptor-ligand model to distinguish the actives and decoys, meaning information provided about the receptors and receptor-ligand binding patterns was not utilized.

**Fig 6:**
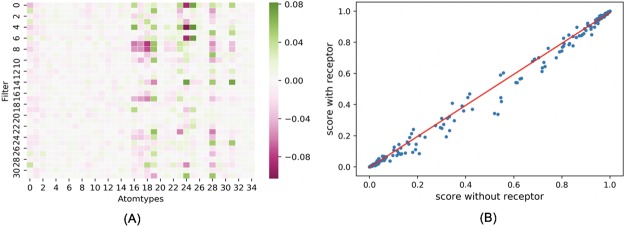
The weights and predicted ligand scores of the AA2AR receptor-ligand CNN model. (A) The average weight put on each atom type in the 32 filters from the first convolutional layer of the AA2AR receptor-ligand CNN model; atom types 0–15 are from the receptor, and atom types 16–34 are from the ligand. (B) Correlation between scores predicted by the AA2AR receptor-ligand CNN model on ligands with vs. without receptor information provided. R^2^ = 0.998.

To further investigate what the receptor-ligand CNN model had learned, we extracted the weights of the 32 filters (3*3*3*35 dimension) from the first convolutional layer of the trained AA2AR receptor-ligand CNN model. As shown in Fig 6A, in the trained model, the weights placed on the receptor (atom type 0 to atom type 15) are much smaller than those placed on the ligand (atom type 16 to atom type 34). Of note, given that in the CNN model there were many layers through which values were transformed nonlinearly, we also compared the ligand scores predicted by the AA2AR receptor-ligand model on the AA2AR ligands with or without receptor information provided. As shown in Fig 6B, the predicted ligand scores were highly correlated (R^2^ = 0.998) between the case when the receptor information was provided in the test set and when it was not, which strongly suggested that the receptor-ligand CNN model did not utilize the receptor information in making its predictions even though the receptor structure was provided during training. Similarly high correlation between ligand scores predicted by receptor-ligand CNN models with or without receptor information provided in the test set was observed for all 102 DUD-E targets (S1 Fig, R^2^ = 0.988).

### Performance of ligand fingerprint-based KNN models

**Fig 7:**
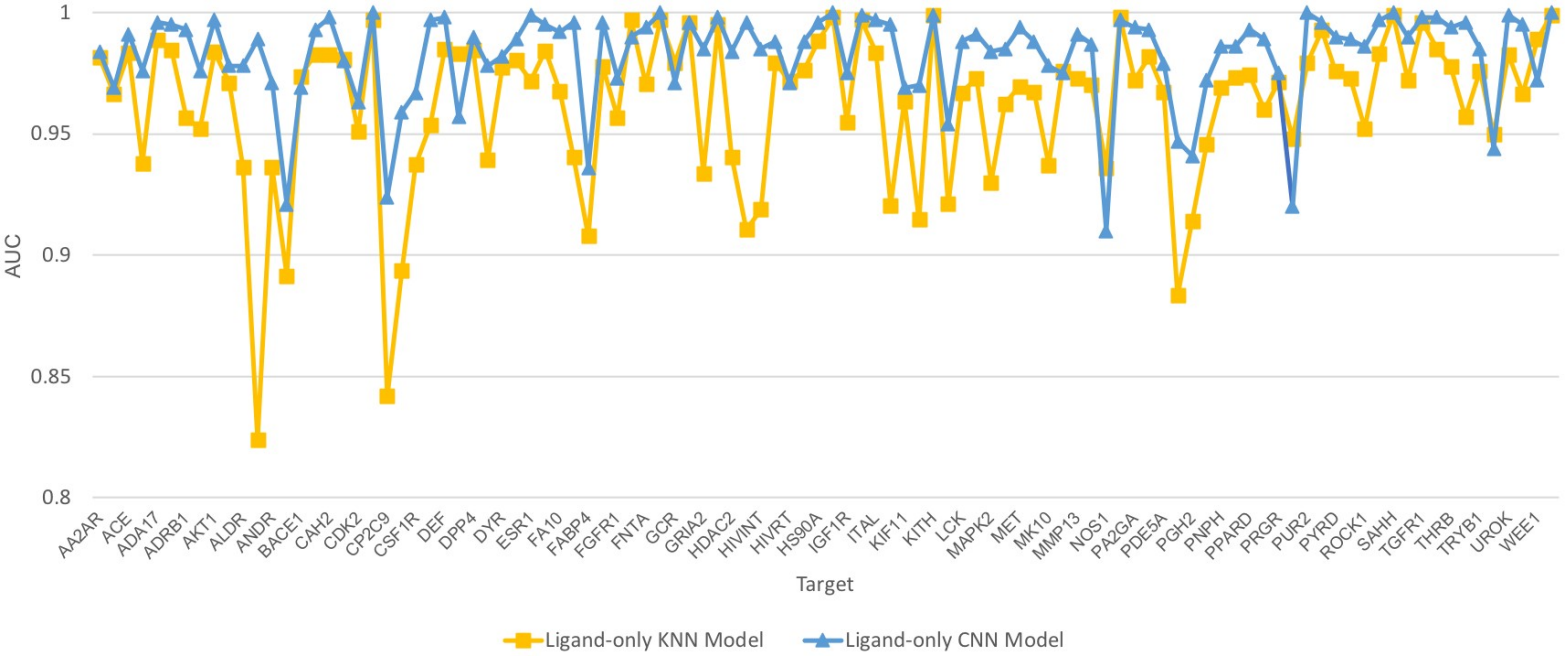
Performance of ligand-trained KNN and CNN models for 102 DUD-E targets.

The high AUC values achieved by the ligand-only CNN model indicated that, for each target, the actives are easily distinguishable from the decoys. To test whether the actives could be distinguished from the decoys using a fingerprint-based feature space, for each target, we calculated ligand fingerprints using the RDKit python package with the default “RDKit” fingerprints in 2048 bits. These fingerprints were then used to build ligand-KNN models where internal cross-validation was used to select the best K values. We then tested these models using the same training and test sets as used for the ligand-trained CNN models. As shown in Fig 7, for all 102 targets, the ligand-KNN models achieved AUC values greater than 0.82, 97 of which were greater than 0.90. It is noteworthy that a simple KNN model performed only slightly worse than a ligand CNN model. In addition, the AUC values from the ligand-only CNN models are moderately correlated (Pearson correlation R = 0.59, average absolute difference 0.02). For example, AUC values that were relatively lower compared to other targets in the KNN models were generally also relatively lower in the CNN models. Further, 96 (94%) targets have a best K equal to 1 or 2, indicating that simple nearest neighbor similarity is highly effective on most DUD-E targets (Table 1). The high performance achieved by the KNN model indicates that, for each target, the actives and decoys are clustered into two separable clusters in the fingerprint-based high dimensional feature space. As the atom type features are correlated to the fingerprint features, the correlated performance between the ligand-based CNN model and KNN model indicates that the high performances of the ligand-only CNN model are attributable to the high similarity among the actives or decoys and distinct separation of these two groups from each other in the feature space.

**Table 1:** The best-K value distribution for 102 ligand-trained KNN models.

K value	Frequency	Percentage
K = 1	79	77.45%
K = 2	17	16.67%
K = 3	4	3.92%
K = 4	0	0.00%
K = 5	2	1.96%
Total	102	100%

### Intra-target analogue bias and decoy bias

**Fig 8:**
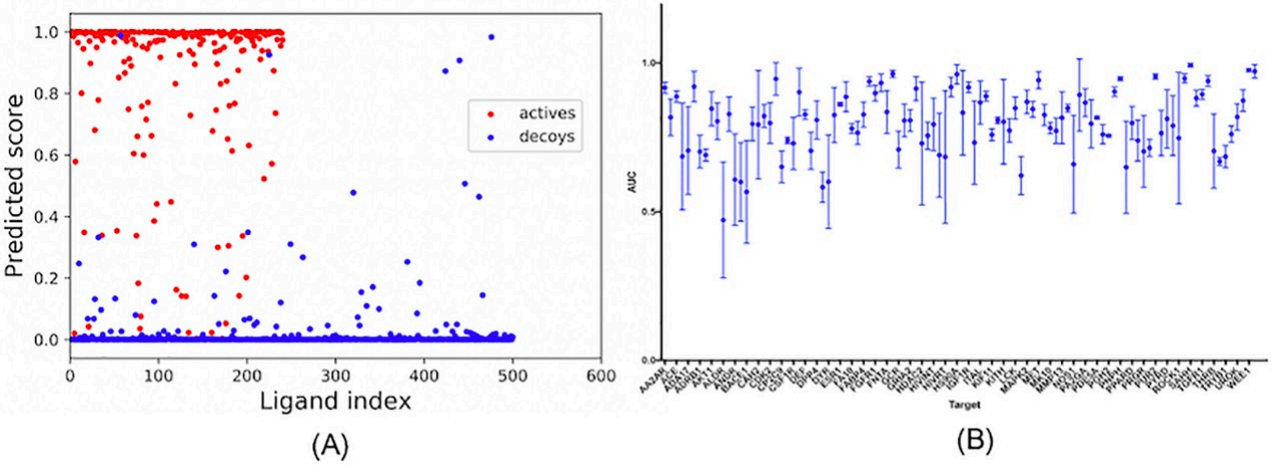
Actives and decoys are generally distinguishable for DUD-E targets. (A) The prediction score of actives and decoys in AA2AR as a representative example; (B) Performance of ligand-trained CNN models trained on small sets of five actives and five decoys. The dots represent mean values, and the bars represent standard deviation.

The high AUC values achieved by the ligand-only CNN model indicated that the actives could be differentiated from the decoys based on the ligand information alone. One possible explanation is that for each target, the actives are analogous, which may lead them to cluster together in the high dimensional space defined by the input representation (analogue bias). In addition, the decoy selection criteria may result in decoys that are easily distinguishable from the actives even in absence of analogue bias (decoy bias). To explore the effects of these biases, we examined the distribution of prediction scores calculated by our ligand-trained CNN models for the actives and decoys. The AA2AR testing set, which had an AUC of 0.98, is a representative example. As shown in Fig 8A, the scores of most actives were higher than those of the decoys, and most of the actives had prediction scores clustered very closely 1, while the majority of the decoys had scores clustered very closely around 0. This score clustering phenomenon was observed for all 102 targets, with the average predicted score for all actives and decoys across all testing sets being 0.90 ± 0.24 and 0.04 ± 0.15, respectively (S2 Fig). Because only ligand information was used to train these models, the highly-clustered nature of the prediction scores for the actives and decoys around 1 and 0, respectively, suggests that the models are learning ligand features that allow them to separate these two groups very well; these may include both analogue and decoy bias.

It is well-accepted that a large training set is required for CNNs to detect patterns and achieve reliable performance. Here, to determine the degree of distinguishability between the actives and the decoys, for each DUD-E target, we randomly selected five actives and five decoys from the previous training set to train the ligand model and then tested the model on the same test set as before. In order to observe how the choice of ligands included in the training set affected the model’s performance, we repeated this procedure three times using different actives and decoys to train the model each time. As shown in Fig 8B, although the training sets were extremely small, the ligand CNN model still achieved high AUC values for many targets (S2 Table), which suggests that the five actives and five decoys in the training sets were able to adequately capture the landscapes of the remaining actives and decoys. The varied standard deviations reflect different levels of analogue and decoy bias for each target. Targets with low standard deviation are likely to have actives and decoys with highly distinguishable features that can be easily extracted from an extremely limited training set, leading the model to successfully separate actives from decoys.

### Inter-target prediction of ligand-trained CNN models

**Fig 9:**
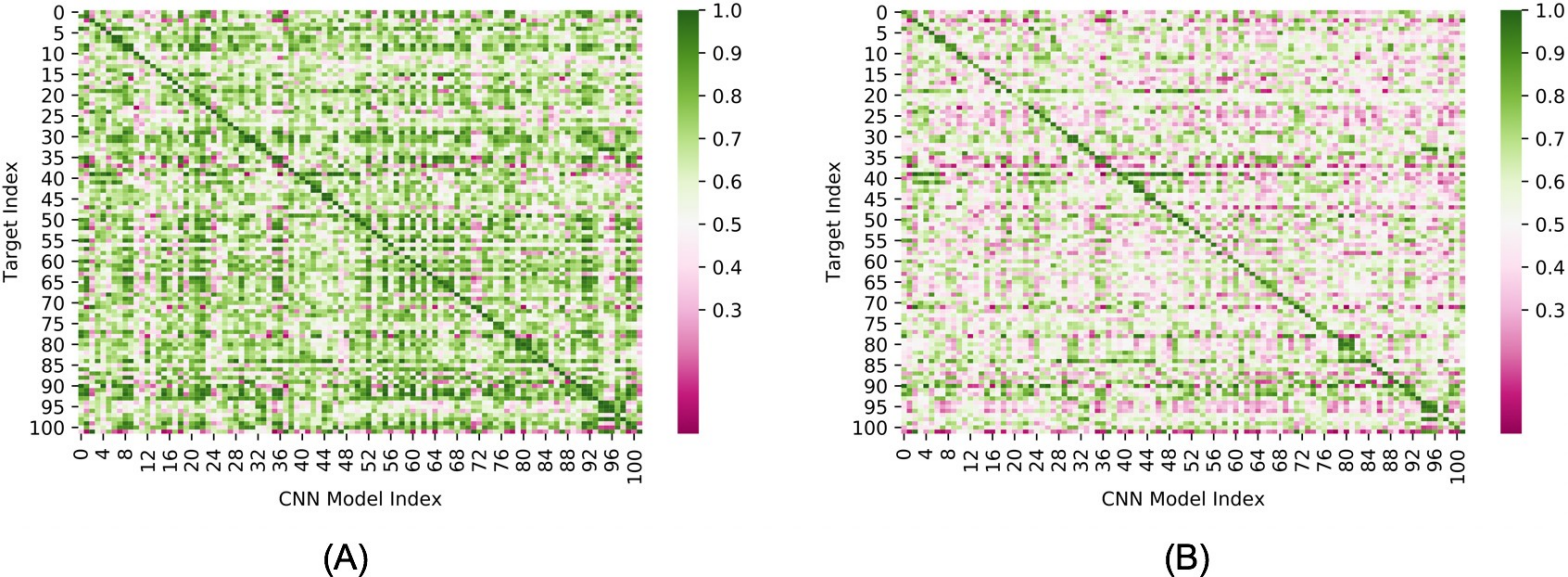
Inter-target prediction performance of ligand-only CNN models. (A) Ligand-only CNN model tested on test sets composed of actives and default decoys (B) Ligand-only CNN model tested on test sets composed of actives and AD decoys. The target order is the same as in DUD-E.

**Fig 10:**
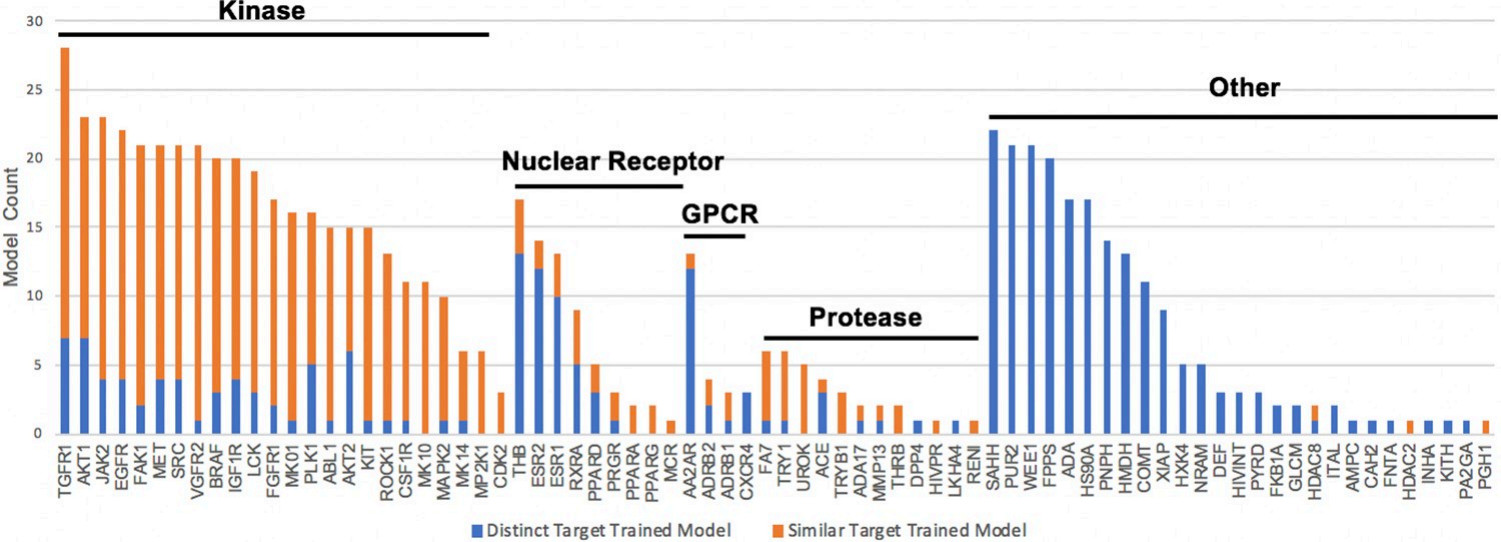
Total number of inter-target models that achieved AUC>0.9 for each target in DUD-E. Targets are partitioned into subsets based on biological families. Targets from a different subset (or non-isoform targets in the "other" subset) are labelled “distinct targets” (blue). Targets in the same subset (except the “other” subset, unless an isoform exists) are labelled “similar targets” (orange). Targets that do not have inter-target high AUC (>0.9) are not shown.

To test a model’s capacity for generalization, many groups have used sequence similarity filters to separate DUD-E targets into diverse training and testing sets. However, this is based on the untested assumption that targets in DUD-E with low sequence similarity have distinct actives. Here, to determine the presence of analogue and/or decoy bias across DUD-E targets, we ran each single-target ligand-trained CNN model against the ligands from all other 101 targets. As shown in Fig 9A, high AUC values not only occurred within targets (diagonal line) but also commonly occurred across targets (AUC values are in S3 Table). For 74 targets, the actives and decoys were accurately distinguished (AUC> 0.9) by one or more models trained on the ligands of other targets (Fig 10). We chose a high AUC value threshold here to ensure that the effects were not due to statistical fluctuations or noise. As expected, models trained by targets within a similar functional category, even those with very low protein sequence similarity, are likely to have high inter-target AUC values. This indicates the sequence similarity threshold is not rigorous enough to exclude bias when constructing training and test sets. For example, actives and decoys for TGFR1 (TGF-beta receptor 1, index = 92) were accurately distinguished by 28 models trained by ligands from other targets (S4 Table). All of these 28 targets plus TGFR1 belong to the category of phosphate-related enzymes, and 24 of them, including TGFR1, are kinases. Of note, these comprise almost all of the 26 kinases present in the DUD-E database. As shown in S5 Table, very few ligands are active against multiple non-isoform targets in the DUD-E. This excludes the possibility that such high inter-target AUC values resulted from different targets having the same actives. This suggests that models trained on kinase targets might have learned shared features of kinase substrates (analogue bias) that makes them perform well for kinase targets in general. However, unexpectedly, high inter-target AUC values frequently occurred for targets that had neither sequence similarity nor shared functionality. As an illustrative example in Table 2 shows that 11 models achieved high AUC (greater than 0.9) values for COMT despite the fact that none of the corresponding targets share significantly similar protein sequence (30%) or functionality with COMT. Inspired by the AVE bias matrix reported by Wallach et al. [38], we calculated the four mean Fingerprint (ECFP4)-based Tanimoto distances between the actives and decoys in the training sets with the actives and decoys in the COMT testing set (training actives to COMT actives, training decoys to COMT actives, training actives to COMT decoys, and training decoys to COMT decoys). We found that these four were similar for all 11 targets and that they were all higher than 0.87 (S3 Fig), which suggests that these high inter-target AUC values do not result from analogue bias. Instead, the models have likely learned features that allow actives and decoys to be easily distinguished (decoy bias).

**Table 2:** Ligand-only CNN models that achieved high AUC (greater than 0.9) for COMT.

Test set	Model trained by	AUC	Sequence similarity*
COMT (Methyltransferase)	ADA (Adenosine deaminase)	0.934	11/21
	CASP3 (Caspase-3)	0.952	No Match
	CP3A4(Cytochrome P450)	0.901	8/23
	DEF (Peptide Deformylase)	0.950	No Match
	GRIA2(Glutamate receptor)	0.926	No Match
	HIVINT (HIV integrase)	0.998	3/8
	HMDH (HMG-CoA reductase)	0.930	11/33
	HS90A (Hot shock protein)	0.994	5/14
	INHA (*Mycobacterium tuberculosis* enoyl reductase)	0.964	8/32
	PPARG (Peroxisome proliferator-activated receptor)	0.951	15/54
	THB (Thyroid hormone receptor)	0.910	No Match

*From the NCBI BLASTp program using the default parameters. x/y is the sequence identity, where x is the number of identical amino acids in the local alignment and y is the total number of amino acids in the local alignment. “No Match” means no alignment was possible for the two sequences. All 11 targets are not homologous to COMT based on a 30% sequence similarity threshold.

The decoy bias in DUD-E results from the criteria for selecting decoys. To remove the contribution of decoy bias to the high inter-target AUC, we constructed the Actives as Decoys (AD) dataset and tested the ligand models on this dataset. As shown in Fig 9B, the number of models yielding a high AUC for each target is significantly decreased (AUC values of AD dataset are in S6 Table, AUC histogram distribution of two datasets is in S4 Fig), which indicates that, for a specific target, models that are trained on the actives of other targets cannot distinguish the actives of that target from the actives of other targets. The fact that the ligand-only CNN model performs well on the default DUD-E dataset but poorly on the AD dataset suggests that, for each target, the ligand-only CNN model learned the biased feature pattern of that target’s decoys, and the model will perform well on other targets if their decoys fit the same biased feature pattern. The decreased performance on AD datasets also occurred when using KNN models (S5 and S6 Figs).

**Fig 11:**
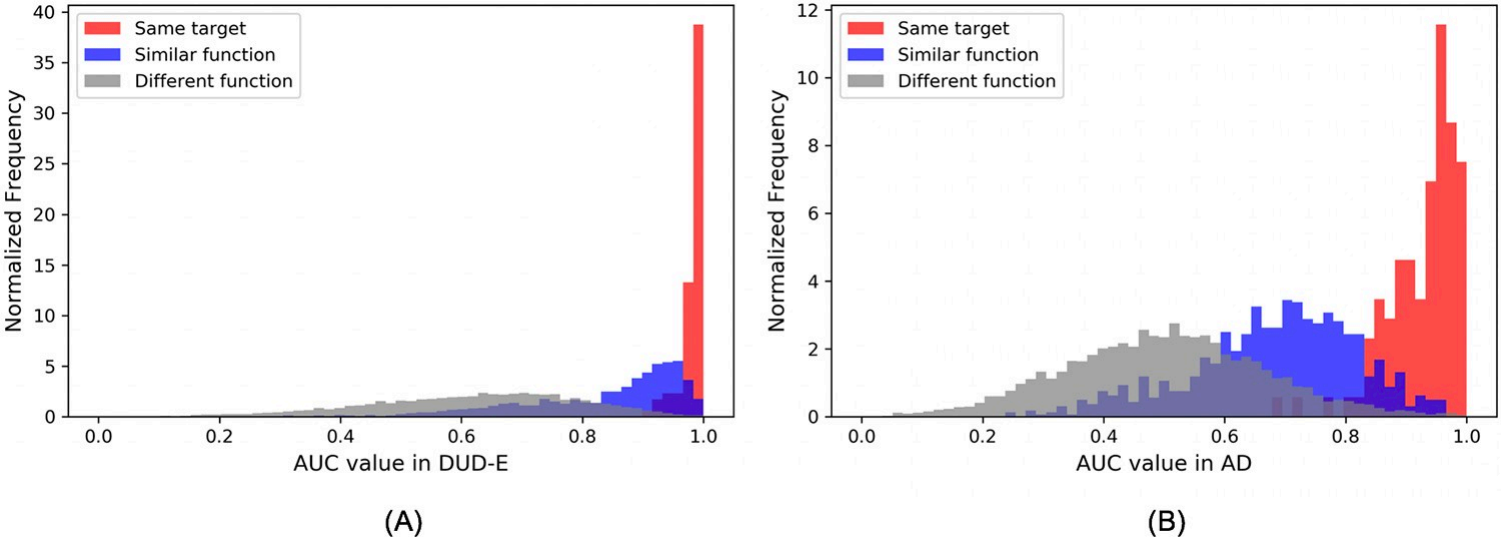
AUC distributions for the ligand-only CNN model (102*102 prediction) across three groups of targets. (A) Results for models tested using the DUD-E dataset; (B) Results for models tested using the AD dataset. The distributions are normalized such that the area under each distribution curve equals 1.

To distinguish intra-target analogue bias, inter-target analogue bias, and decoy bias in the performance of the ligand-only CNN model, we categorized the 102 * 102 AUCs (Fig 9) into three groups: 1) A same target group (red in Fig 11), in which the models used for prediction were trained and tested on the same target; 2) A similar function group, in which the models used for prediction were trained on proteins with a similar function as the test set. The functional groups for this were: kinases, proteases, GPCRs, and nuclear receptors. (blue data) 3) A different functional group, in which the models used for prediction were trained by targets with different functionality than the proteins in the test set (grey data). Table 3 shows the average AUC for these three groups and Fig 11 shows the AUC distribution for each group for the both the DUD-E dataset and to the AD dataset.

**Table 3:** The mean and SD of the AUC values across three target groups.

		Same Target	Similar Function	Different Function
DUD-E dataset	Mean	0.983	0.835	0.618
	SD	0.019	0.133	0.176
AD dataset	Mean	0.927	0.682	0.500
	SD	0.060	0.137	0.165

The AUC values for the “same target group” were the highest on average for both the DUD-E and AD datasets. We attribute this high performance regardless of dataset to intra-target analogue bias which is consistent with the conventional wisdom that ligands that bind to the same target have chemical similarities. Similar results are seen in the blue curves which we attribute to inter-target analogue bias which is consistent with general belief that compounds that bind to proteins with similar function often have similar chemical features. As expected the inter-target analogue bias is less than the intra-target analogue bias.

The average AUC values for all three groups are lower for the AD dataset than for the DUD-E dataset (Table 3 and S7 Fig). We attribute these differences to the contribution of decoy bias to the model performance as the AD dataset was designed to eliminate the decoy bias that was introduced by the DUD-E decoy selection criteria. In this work, we proposed that the CNN models could learn from three different sources: 1) protein-ligand interaction 2) analogue bias and 3) decoy bias. Since the receptor information is absent from the ligand-only model, it could not learn from the first source. The analogue bias and decoy bias were controlled for in the “different function” group in the AD dataset, we therefore expect that the model would perform randomly on this group, and, indeed, the average AUC for the “different function” group in AD dataset was 0.500.

### Multi-target CNN model

**Fig 12:**
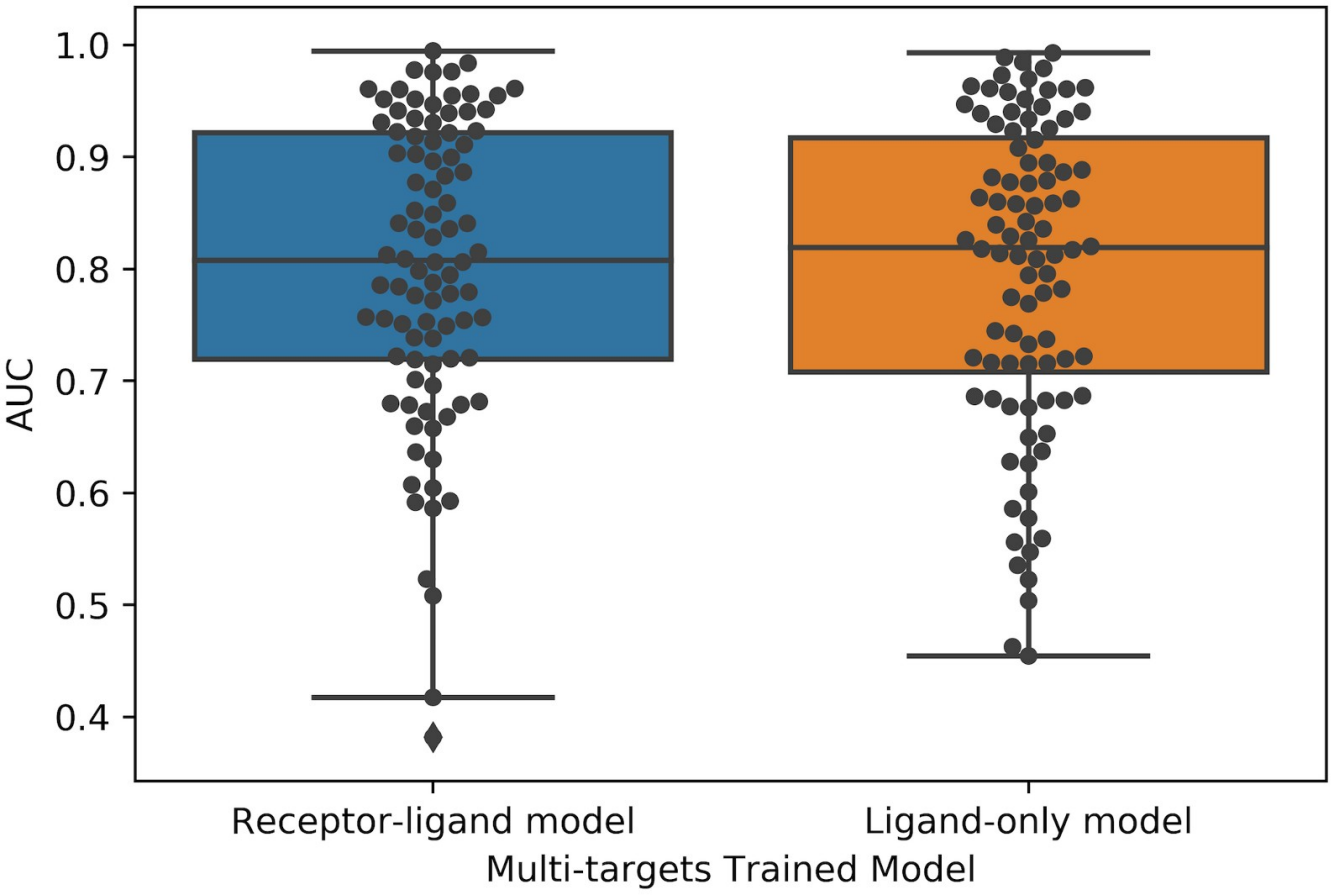
Comparison of the performance of multi-target trained receptor-ligand CNN model and ligand-only CNN model. The two models were trained on 10 targets and tested on the remaining 92 targets. The receptor-ligand CNN model was trained on receptor-ligand 3D binding poses, and the ligand-only mode was trained on ligand binding poses alone. Each black dot represents a target.

To investigate whether a CNN model trained on a subset of targets could be applied successfully to a new target, we trained the receptor-ligand and ligand-only CNN models on a training set of ten targets (AA2AR, CXCR4, FA10, FABP4, GLCM, HIVPR, ITAL, KIT, KITH and LCK) and then applied these two models to the remaining 92 DUD-E targets. As shown in Fig 12, the receptor-ligand and ligand-only CNN model showed similar performance (both with AUC 0.80 ± 0.13) when tested on the remaining 92 targets. To investigate whether the receptor information was utilized by the receptor-ligand model when trained by multiple targets, we also applied the model to a testing set wherein the receptor was replaced by a dummy atom. As shown in S8 Fig, the receptor information was not utilized in most cases. We also tested the multi-target-trained ligand-only model and receptor-ligand model on AD datasets. As shown in S9 and S10 Figs, the AUCs shifted downward for all targets, and the average performance was similar to that of random chance.

### Performance of CNN models trained on the PDBbind database

**Fig 13:**
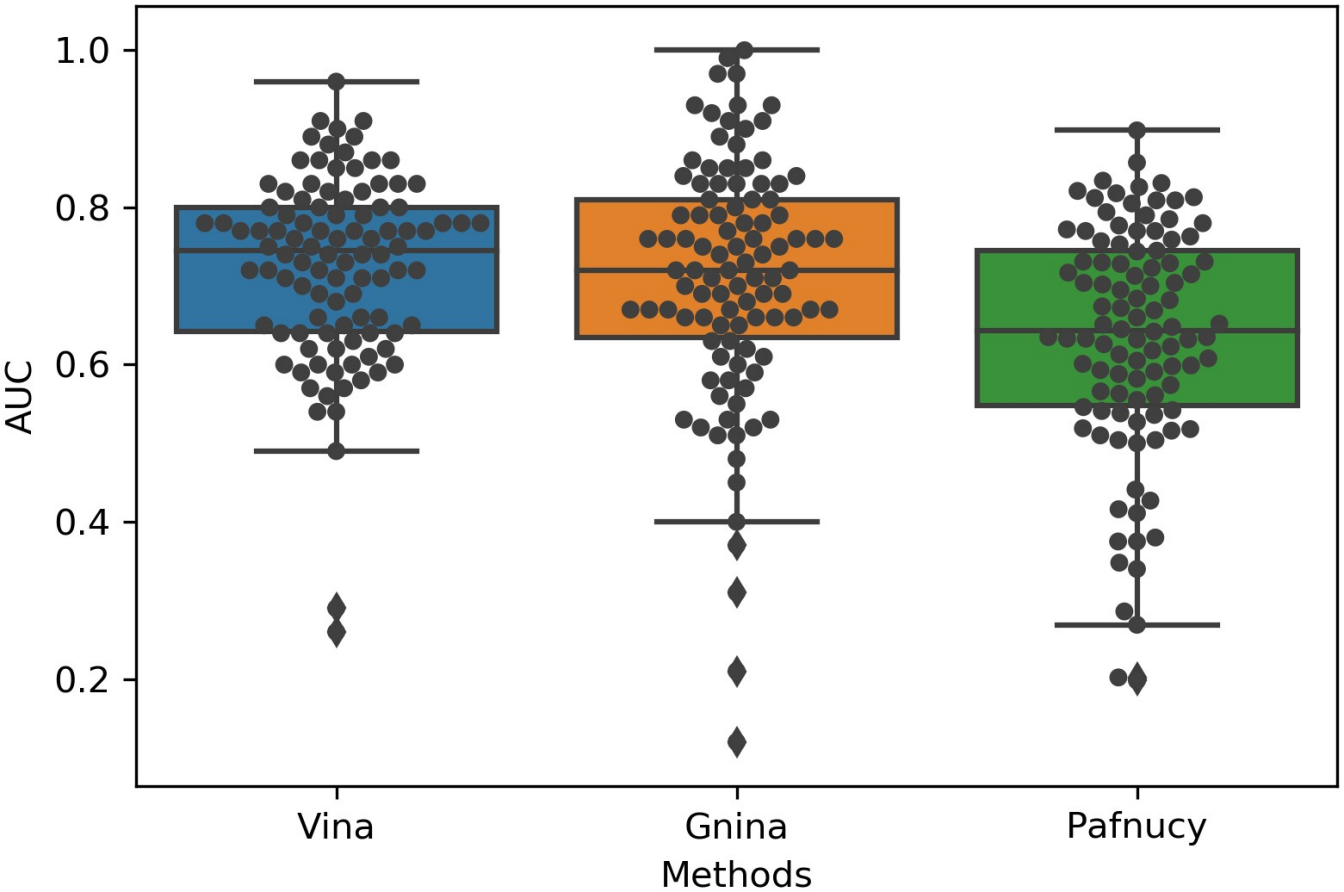
The AUC value distribution for Vina, Gnina and Pafnucy performed on all 102 DUD-E targets. Each black dot represents a DUD-E target.

Apart from categorical prediction, many recent studies [19–21,26,41] have also showed that deep learning models trained on the PDBbind [49] “refined” set can predict binding affinity in the “core” set to a Pearson correlation coefficient of ~0.8 or root-mean square deviation (RMSD) < 2 in terms of pKi or pKd. However, as Yang et al. [50] showed, sequence similarity has a significant effect on the performance of machine learning-based scoring functions. Therefore, the fact that the core set overlaps with the refined set even when the core set items are removed from the refined set could make the reported performance over-optimistic. Here, we tested two previously-trained open-source structure-based CNN models, the Gnina model [41] and the Pafnucy model [21], on all 102 DUD-E targets and compared their performance with that of Vina. Briefly, the Gnina model was trained by Hochuli et al. [41] on docked poses from the PDBbind refined set; poses that were within 2 Å RMSD of the crystal structure were assigned the same binding affinity as the crystal pose, while poses that had RMSD values greater than 4 Å from the crystal structure were trained using a hinge loss that only penalized over-prediction of the associated affinity. In contrast, the Pafnucy model was trained by Dziubinska et al. [21] on a “filtered refined set” of protein-ligand crystal structure data constructed by removing the core set from the PDBbind refined set. Since the Pafnucy model was trained on protein-ligand crystal structures that the ligands in DUD-E do not have, we fed both models with the top nine docked poses, as studies [6,51] have shown that the probability that a successful pose RSMD (< 2 Å) is present within the top three poses is high (~80%). In each case, the top ranked pose was used to score a given ligand. As shown in Fig 13, the performance of these three models varies from target to target. As summarized in Table 4, Vina performed comparably to Gnina, and they both performed better than Pafnucy.

**Table 4:** Summary of Vina, Gnina and Pafnucy performance on DUD-E targets.

	Average AUC	Frequency (AUC>0.8)	Frequency (AUC>0.9)
Vina	0.725	24	3
Gnina	0.709	28	10
Pafnucy	0.632	12	0

### Pose sensitivity

**Fig 14:**
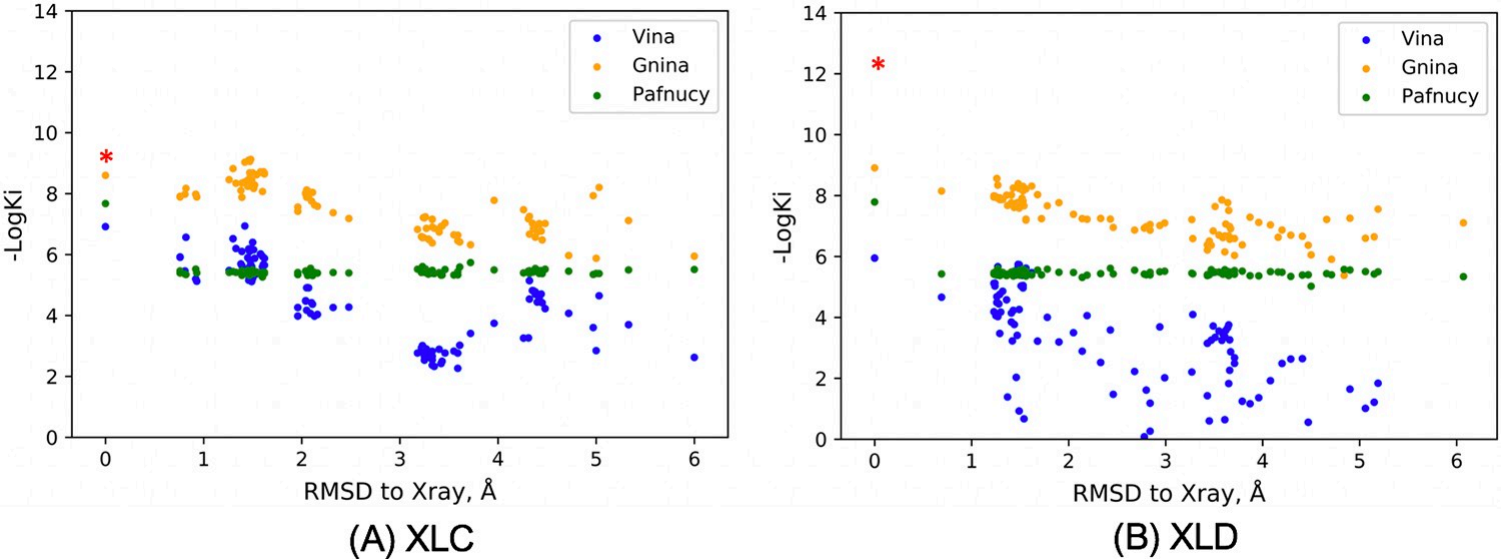
Pose sensitivity of Vina, Gnina and Pafnucy. The three models were tested on 100 re-docked poses of ligand XLC (A) and ligand XLD (B). The red asterisk at the RMSD = 0 marks the experimental affinity. Vina predicts free binding energy (ΔG) in kcal/mol; here, we estimated the Ki at 25 Celsius using the equation ΔG = RTlnKi, where R is the gas constant (8.31 J/K·mol).

It is generally thought that models that can learn protein-ligand binding patterns will gain the ability to generalize which can, in turn, lead to good prediction of actives for a wide range of targets. However, our results have shown that good prediction in a test set does not necessarily mean the model has learned physical binding patterns. To further investigate whether the open-source structure-based CNN models have learned meaningful features from binding patterns, we tested Gnina and Pafnucy’s performance on Human blood coagulation Factor Xa (FXa). FXa is a drug target for anti-coagulation therapy and a series of compounds with different levels of binding affinity have been synthesized, which provides a dataset to assess the scoring function’s sensitivity to ligand chemical components [52,53]. Among these compounds, XLC (PDB ID: 1MQ5) and XLD (PDB ID: 1MQ6) are two chemically-similar ligands with high-quality crystal structures, and their binding affinities were determined to 1 nM for XLC and 7 pM for XLD, respectively [54]. To evaluate the pose sensitivities of Gnina and Pafnucy, we re-docked each ligand to the binding pocket to generate 100 different poses with root mean squared distance (RMSD) of heavy atoms ranging from 0.0–6.0 Å. As shown in Fig 14, for Vina and Gnina, although the chemical components of the ligands are same, different binding affinities were predicted. In contrast, for Pafnucy, except for the fact that the crystal poses were predicted to have a different binding strength, all other poses were predicted to have nearly identical affinity even when the RMSD was large. This may be because the Gnina model was trained by sets of docked poses for each ligand, among which “crystal-like” poses were assigned good affinity while poses less similar to the crystal-like pose were assigned lower affinity. On the other hand, the Pafnucy model was trained only by crystal poses, which may lead the model to be insensitive to pose change. All three methods all failed to distinguish the affinity difference between XLC and XLD, indicating accurate binding affinity prediction for similar ligands remains a challenge.

## Conclusions

In this study, we showed that the performance of protein-ligand CNN models is affected by hidden biases in the DUD-E dataset. We showed that analogue biases are common both within the sets of actives associated with each target (intra-target analogue bias) and across sets of actives associated with different targets (inter-target analogue biases). We further provided evidence for the existence of decoy bias likely resulting from the selection criteria used during construction of the DUD-E dataset. Analogue bias and decoy bias allow CNN models to learn entirely from the ligands even though protein structure information for the target is provided during the training stage. We also tested additional CNN models trained by protein-ligand crystal structure data from PDBbind. Although these models were reported to have good performance in their test datasets, they did not outperform the docking program Autodock Vina on average when tested using DUD-E. Our studies suggest that 1) DUD-E should be used with extreme caution when applied to machine learning-based method development and ideally should be avoided and 2) rigorous testing of a model’s response to different types of information in training/test sets is essential to building a meaningful model.

## Discussion

As deep learning methodologies have been increasingly applied to virtual screening, our study suggests that caution should be taken as hidden bias may exist in datasets used to develop these methods. We have shown evidence for both analogue and decoy bias in the DUD-E dataset. Analogue bias most likely originates from the fact that ligands binding to a specific target (or to a set of targets with similar functionality) are likely to have similar scaffolds, resulting in similar topological features that are easily captured by CNN architectures. Decoy bias in DUD-E was introduced by the criteria that were used to select the decoys for each target. For example, in DUD-E, to control the false decoy rate, for each active, candidate decoy compounds were sorted by the topological fingerprint-based Tanimoto Correlation, and the top 75% were removed, leaving only the 25% most dissimilar compounds as decoys for that active [37]. Although these criteria minimize the false decoy rate, they also cause the decoys to be easily distinguishable from the actives by machine learning models. Also, Sieg et al. [55] recently published an analysis of the molecular and physical properties of the actives and decoys from DUD-E, which showed that certain properties are exclusive to one group or the other. For example, compounds with molecular weight greater than 500 Da include actives only. They also suggested these simple distinguishable features between actives and decoys allow machine learning-based models to distinguish DUD-E actives from decoys on the basis of the ligands themselves, which is consistent to our findings. Together, the biased basic properties of the ligands and overly-conservative selection criteria may also result in overall separation of the decoys and actives in the high-dimensional space constructed by the combination of all their features such that models can distinguish the non-binders from binders in general but cannot tell which target each binder associates with.

Besides bias, there are many additional obstacles that lie on the road to successfully applying deep learning to virtual screening. One is data quality. In the image recognition domain, humans can easily recognize images in any number of different contexts; for example, we perceive automatically that two pictures of a cat in which the cat’s tail has shifted positions are still both of a cat. As a result, humans can provide vast amounts of high-quality data to train image recognition models. Unfortunately, without expert knowledge, we do not know whether a small shift of a chemical group will affect a compound’s ability to bind to a target with the same level of affinity. This introduces uncertainty into the quality of pose data that is fed into binding prediction models when docked poses are used as training input. Another challenge is data paucity. Current deep learning models can easily have more than 30 atom type channels, significantly more than image recognition models, which only have three channels. The increased dimensionality exacerbates the paucity of protein-ligand crystal structure information, and the millions of parameters-much more than the current number of available data points-encourages the model to simply memorize the entire set of data points, complicating generalization to novel compounds [56]. In summary, low data quality and data paucity together make it a very challenging task to develop a deep learning model for binding affinity prediction that can generalize to new protein targets and different ligand scaffolds. Here, we also showed that high performance in test sets is not enough to make the model generally applicable, as hidden biases may exist in the training/testing datasets that can lead the model astray. To ensure that models have learned meaningful features, we should test them by interrogating their response to different types of training or testing information and ensuring their sensitivity to ligand binding pose.

In this work we have introduced controls in datasets to test whether a model is learning from protein-ligand interactions, analogue bias or decoy bias. By removing receptor information from the test set for receptor-ligand models, we can determine how much the model is learning from the receptor and hence from protein-ligand interactions. Similarly, testing on a dataset that does not share decoy bias introduced by the decoy selection criteria (as we did with the AD dataset) helps identify how much a model is learning from decoy bias. Inter-target validation on test sets from which proteins that share homology and functional similarity with training set proteins have been removed controls for real analogue bias and constructed decoy bias. These tests should be expanded upon and refined in the future and be broadly applied to machine learning outcomes to ensure that the machine learning black box is learning from meaningful information that is generalizable to making prospective predictions on molecular recognition. In this study, we highlighted the danger of attributing a model’s high performance in a test set to successful generalization of binding interactions without rigorously validating the model. Although many machine learning-based methods have been developed and tested on DUD-E [23–25,27,32,35], we clearly showed here that analogue bias and decoy bias are widespread in DUD-E and, consequently, models may only learn the inherent bias in the dataset rather than physically meaningful features. We hope this work can help our community become more aware of the pitfalls of current databases and develop more robust and meaningful deep learning models for drug discovery.

## Supporting information

S1 Fig Correlation of ligand scores predicted by the receptor-ligand CNN model with vs. without the receptor provided in the test set for all 102 DUD-E targets. (TIFF)

S2 Fig The distribution of the prediction score of all actives and decoys from the 102 DUD-E target test set. (TIFF)

S3 Fig The average fingerprint-based Tanimoto distance between the actives and decoys from training sets and COMT test set. The ligand-only models trained by these 11 targets all achieved high AUC in COMT.
(TIFF)

S4 Fig The distribution of AUC values achieved by ligand-only CNN models tested on the default DUD-E dataset and the AD dataset. In the default dataset, the decoys are the DUD-E decoys, while in the AD dataset, the AD decoys are the actives from other targets.
(TIFF)

S5 Fig The KNN model performance on the default DUD-E dataset and the AD dataset. (TIFF)

S6 Fig The distribution of AUC values achieved by the KNN model tested on the default DUD-E dataset and the AD dataset. (TIFF)

S7 Fig The distribution of AUC values in each target group using DUD-E and AD dataset. (TIFF)

S8 Fig Performance of the receptor-ligand model for the same ligand test sets with and without receptor information. For each target, red dots indicate performance when the receptor structure was provided in the test set, while blue triangles indicate performance when the receptor structure was replaced by a single dummy atom.
(TIFF)

S9 Fig Multi-target trained ligand-only models tested on 92 targets with default decoys and AD decoys. The average AUCs of the default and AD testing sets are 0.80 and 0.53, respectively.
(TIFF)

S10 Fig Multi-target trained receptor-ligand model tested on 92 targets with default decoys and AD decoys. The average AUCs of the default and AD testing sets are 0.80 and 0.54, respectively.
(TIFF)

S1 Table Atom types used in our CNN model. There are 35 atom types (channels) in total: the first 16 channels are from the receptor, and the remaining 19 channels are from the ligand.
(XLSX)

S2 Table AUC values achieved by the ligand-only CNN models trained by a small number of actives/decoys for each target. Replication n = 3.
(XLSX)

S3 Table AUC values for the 102*102 cross-target prediction by ligand-only CNN models tested on the DUD-E dataset. (XLSX)

S4 Table The ligand-only CNN models that achieved high AUC for TGRF1 and the functionality of the targets that the models were trained on. (XLSX)

S5 Table Active overlap among the 102 DUD-E targets. (XLSX)

S6 Table AUC values for the 102*102 cross-target prediction by ligand-only CNN models tested on the AD dataset. (XLSX)
