# Supplementary figures and images for: Hidden bias in the DUD-E dataset leads to misleading performance of deep learning in structure-based virtual screening
Source: PLoS One. 2019 Aug 20;14(8):e0220113. doi: 10.1371/journal.pone.0220113 (PMC6701836; doi:10.1371/journal.pone.0220113)

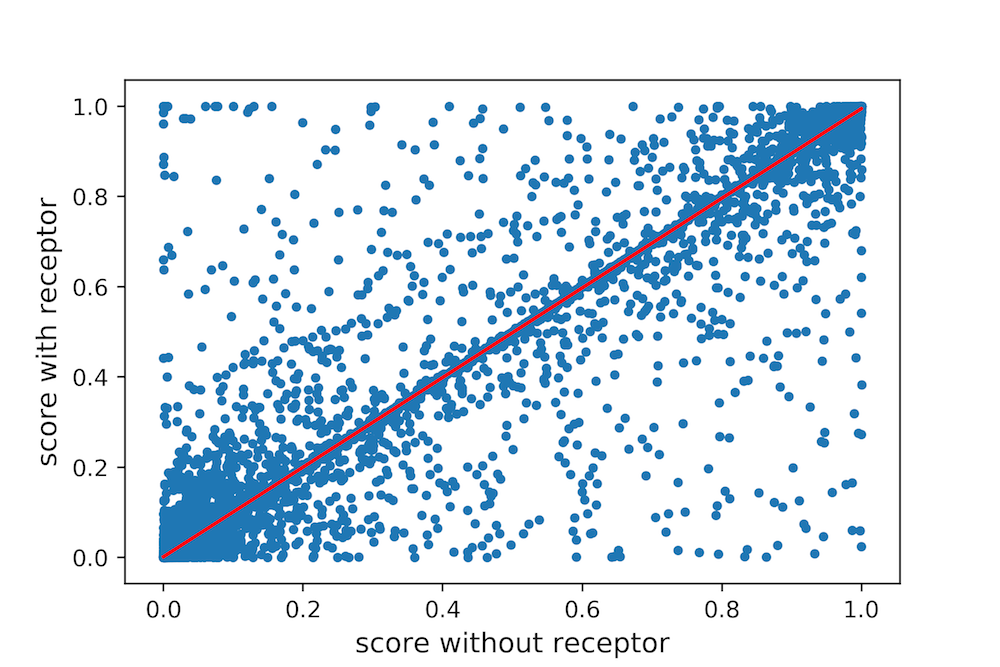

Supplement: S1 Fig — (TIFF) [file pone.0220113.s001.tiff]

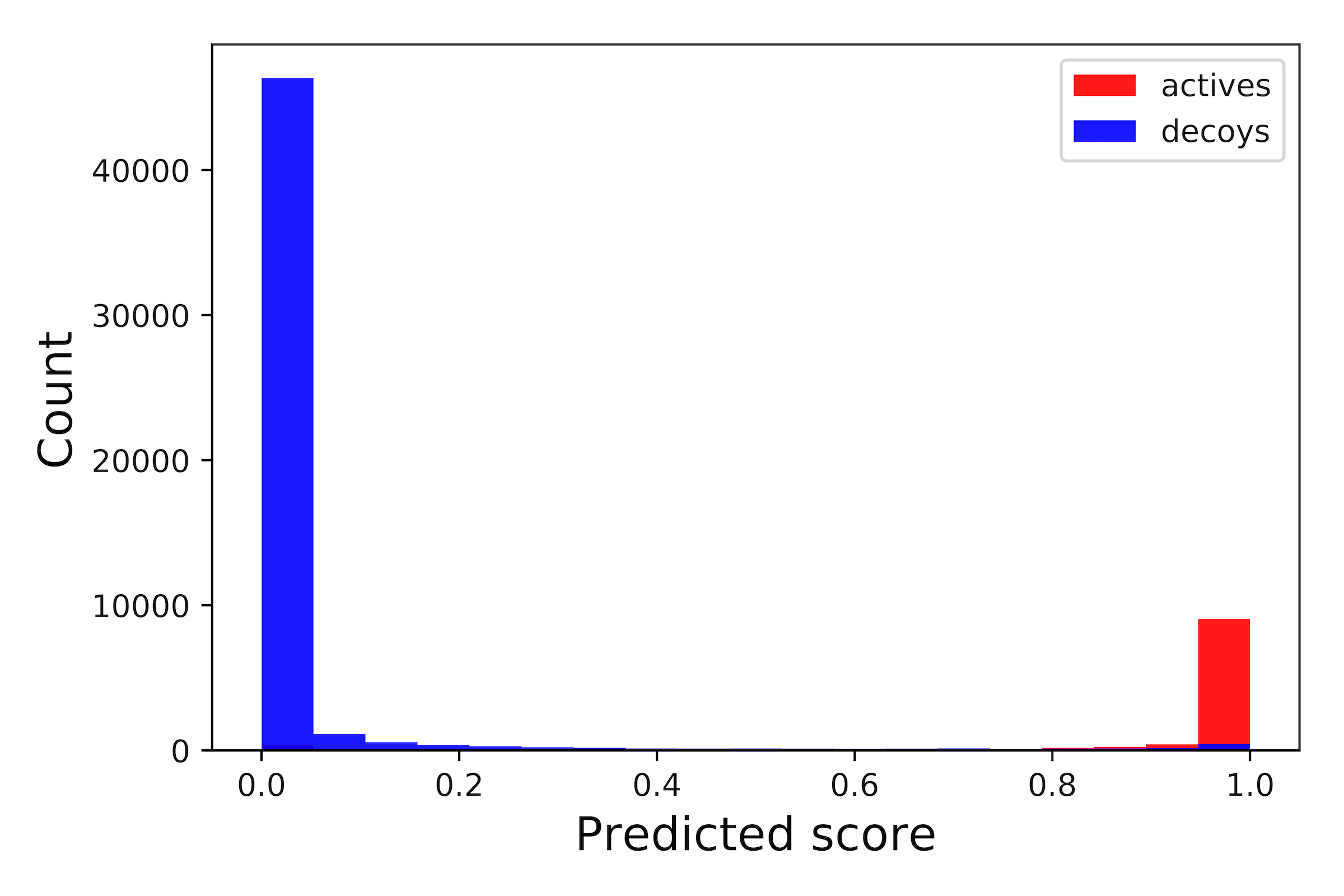

Supplement: S2 Fig — (TIFF) [file pone.0220113.s002.tiff]

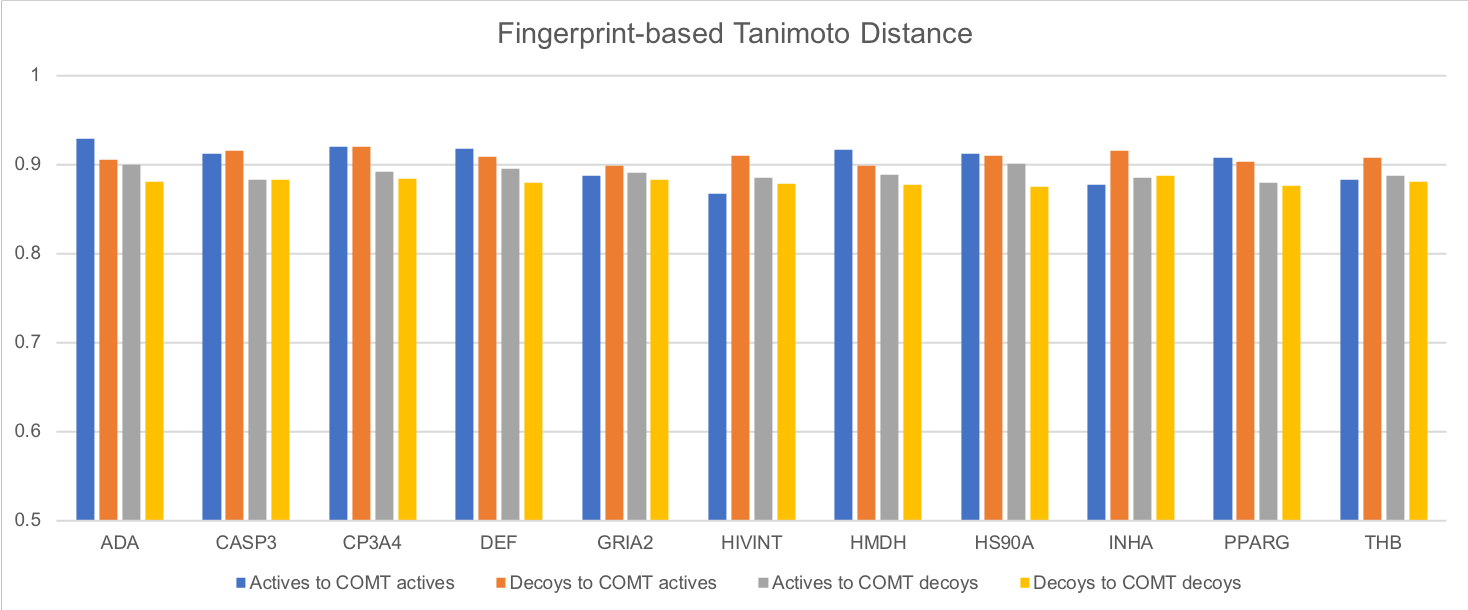

Supplement: S3 Fig — The ligand-only models trained by these 11 targets all achieved high AUC in COMT. (TIFF) [file pone.0220113.s003.tiff]

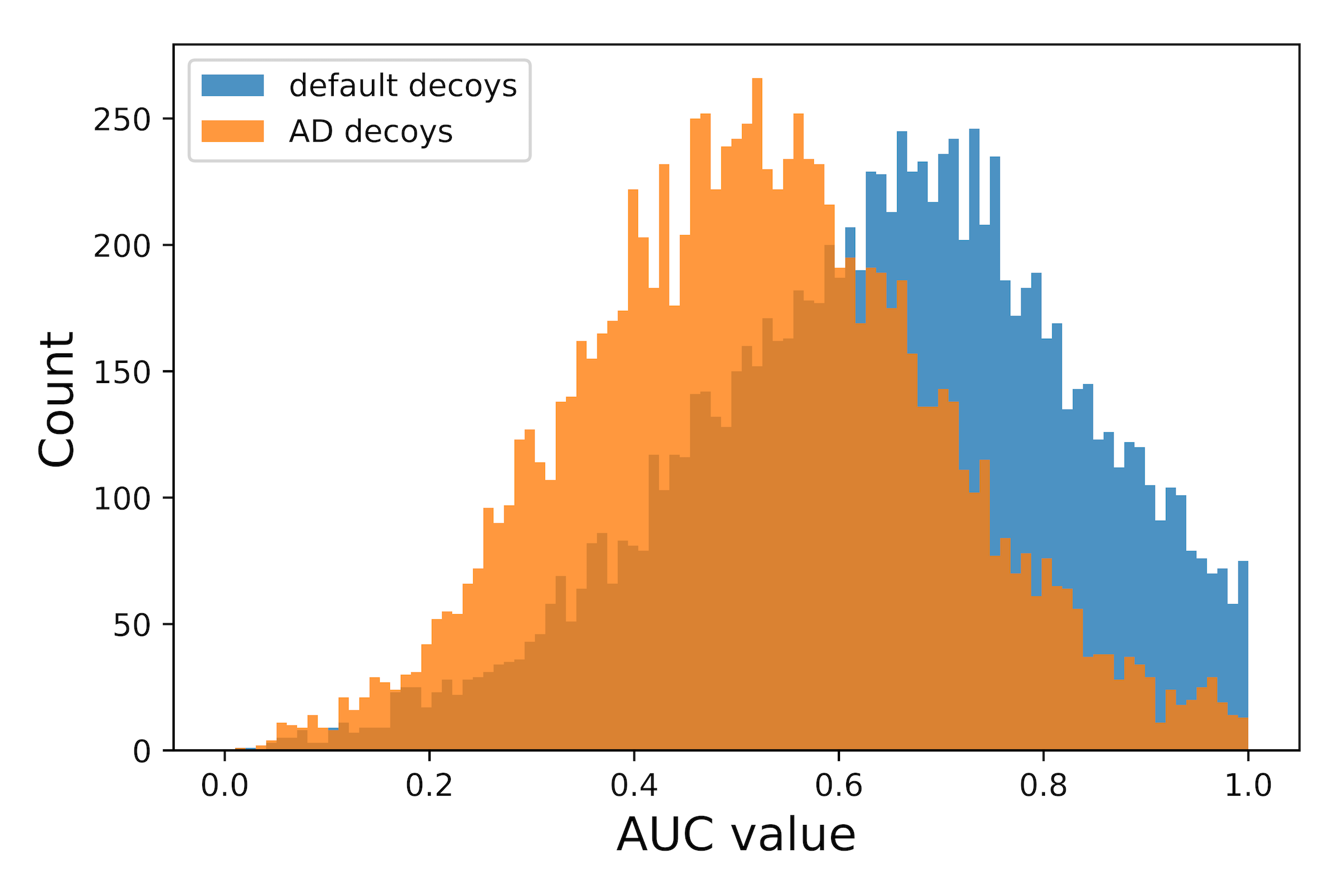

Supplement: S4 Fig — In the default dataset, the decoys are the DUD-E decoys, while in the AD dataset, the AD decoys are the actives from other targets. (TIFF) [file pone.0220113.s004.tiff]

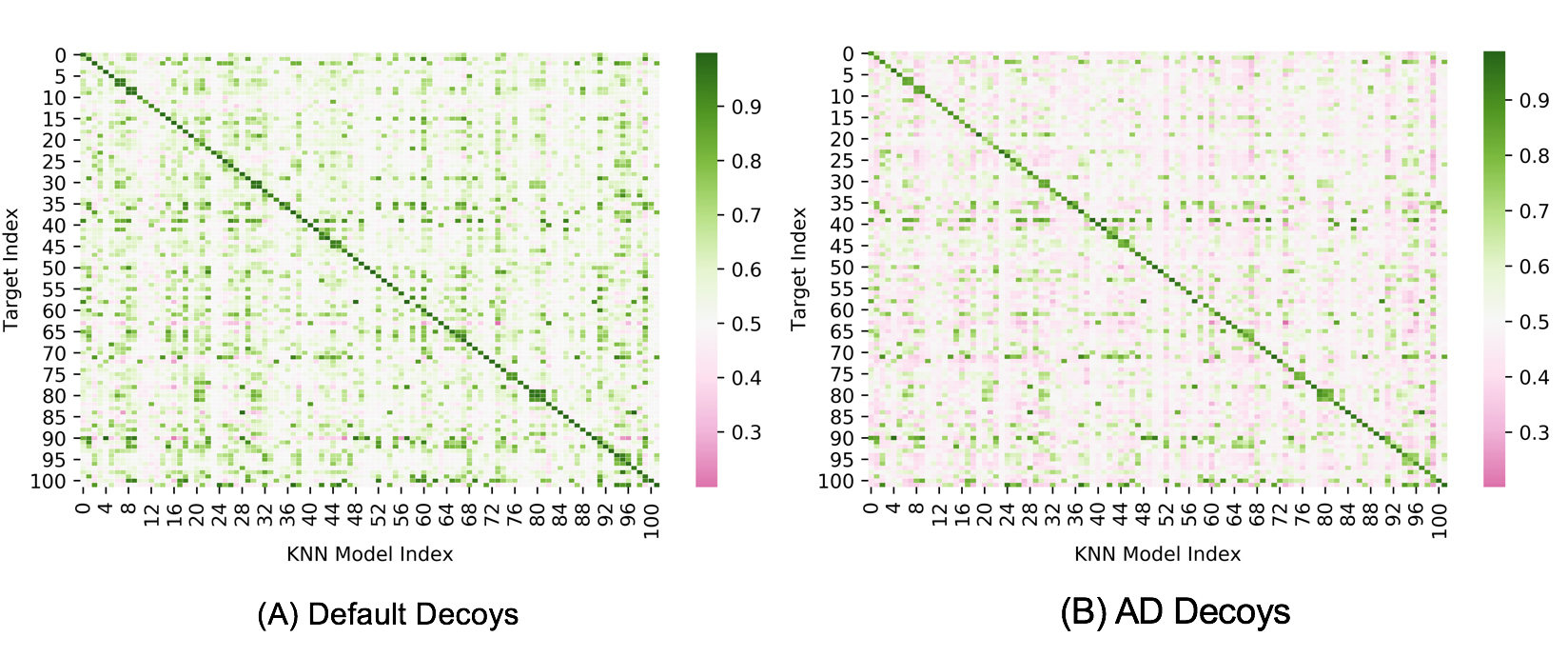

Supplement: S5 Fig — (TIFF) [file pone.0220113.s005.tiff]

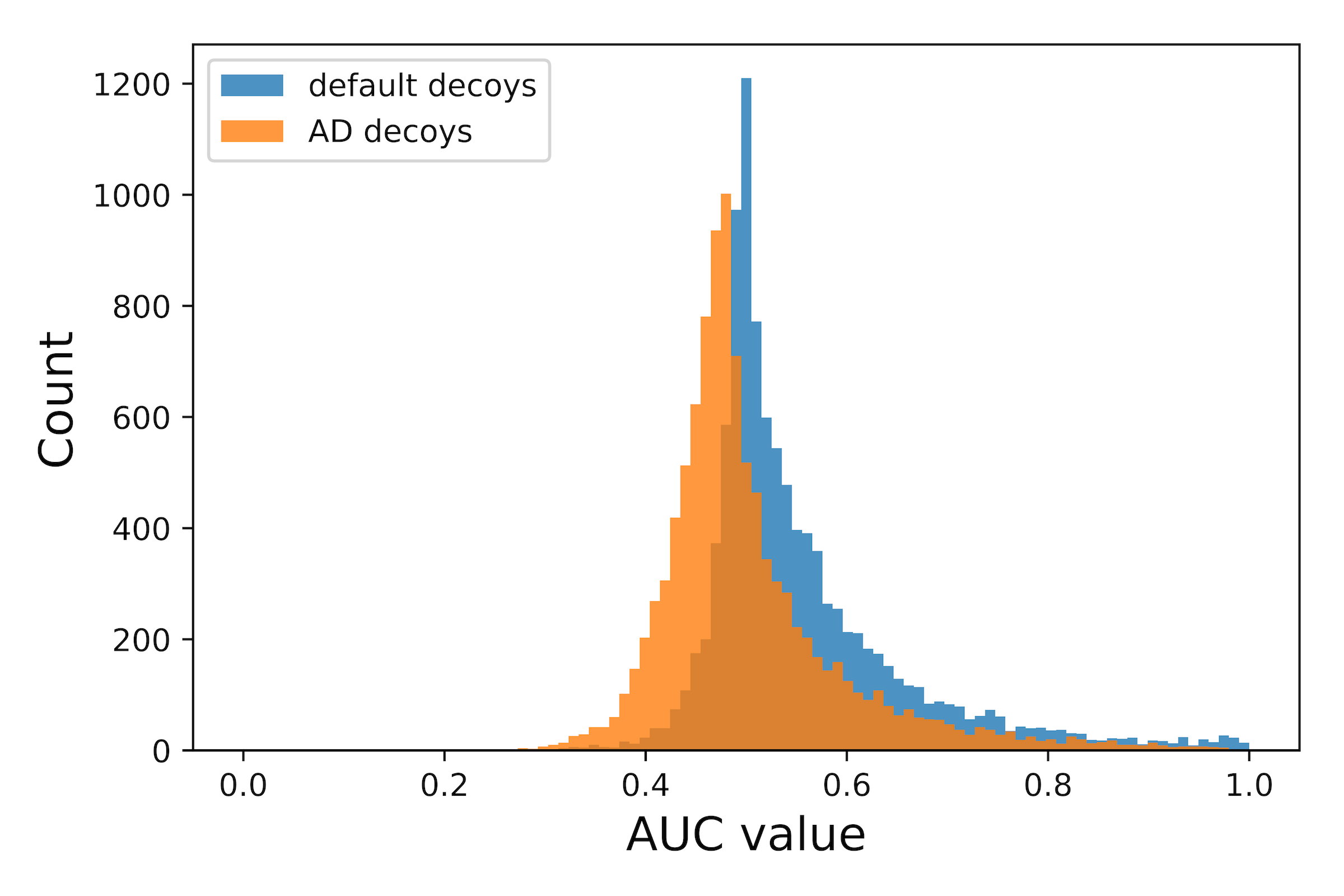

Supplement: S6 Fig — (TIFF) [file pone.0220113.s006.tiff]

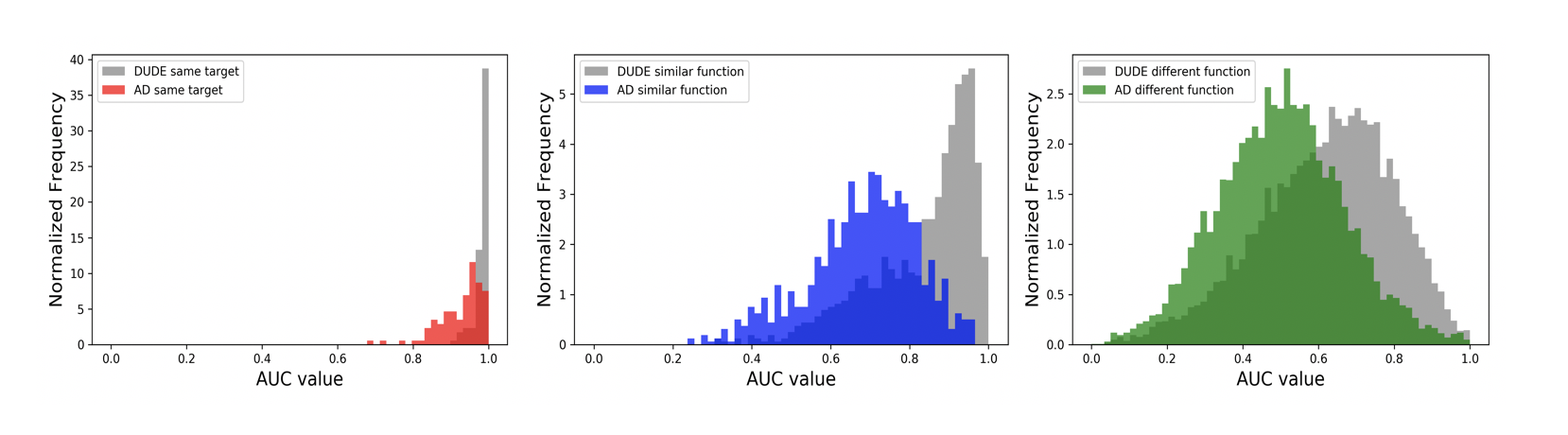

Supplement: S7 Fig — (TIFF) [file pone.0220113.s007.tiff]

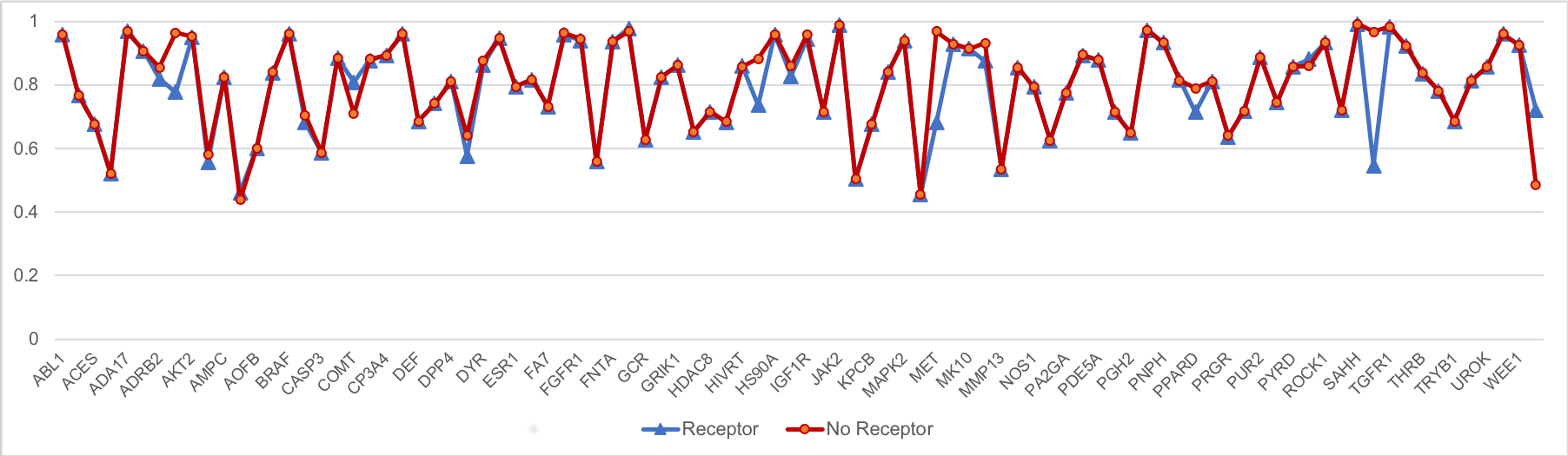

Supplement: S8 Fig — For each target, red dots indicate performance when the receptor structure was provided in the test set, while blue triangles indicate performance when the receptor structure was replaced by a single dummy atom. (TIFF) [file pone.0220113.s008.tiff]

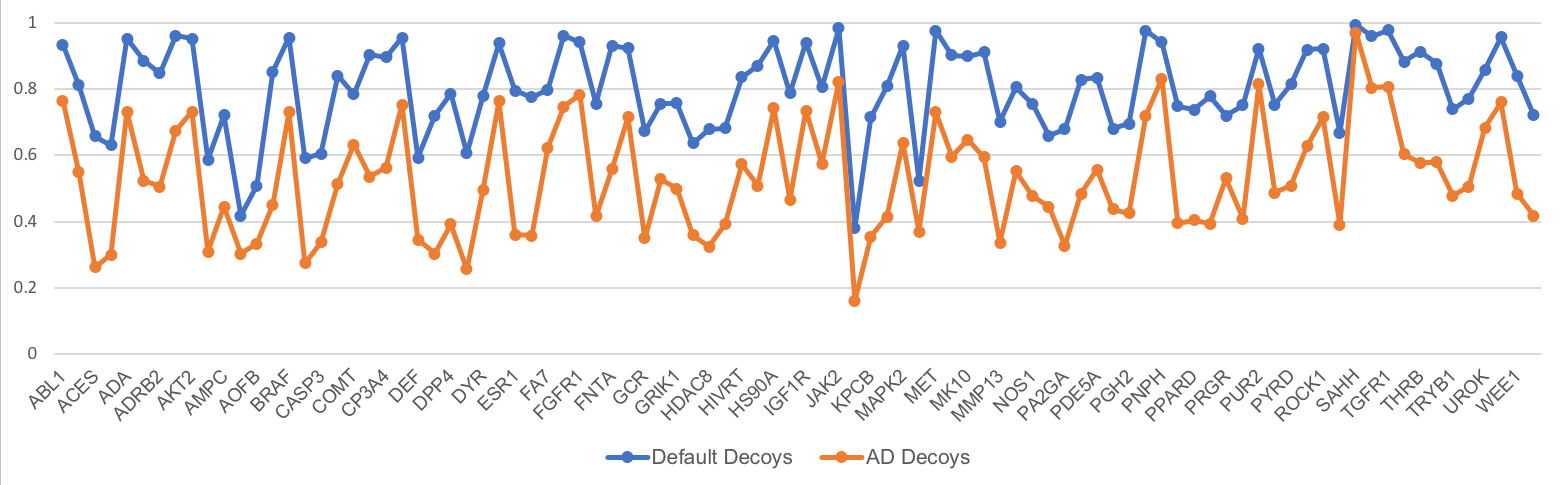

Supplement: S9 Fig — The average AUCs of the default and AD testing sets are 0.80 and 0.53, respectively. (TIFF) [file pone.0220113.s009.tiff]

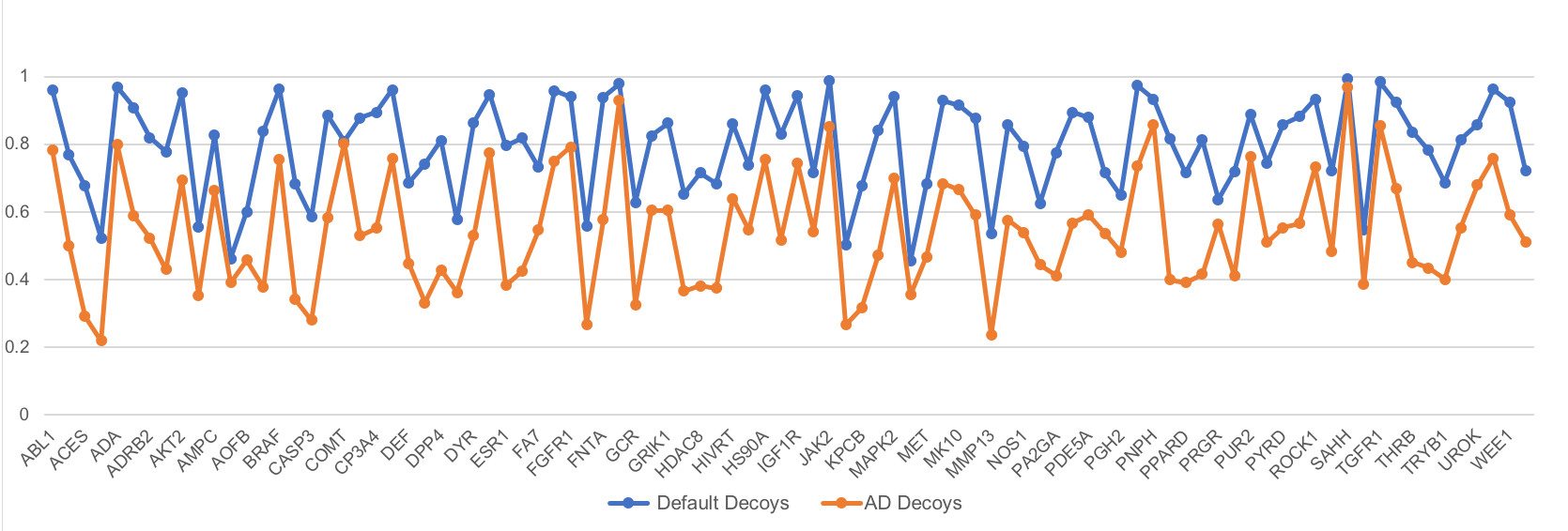

Supplement: S10 Fig — The average AUCs of the default and AD testing sets are 0.80 and 0.54, respectively. (TIFF) [file pone.0220113.s010.tiff]
